# Perceptions of care home residents, families and staff about wearable devices: a scoping review

**DOI:** 10.1007/s41999-026-01550-7

**Published:** 2026-07-10

**Authors:** Ana Swain-Velasco, Kirsty Hynes, Susan D. Shenkin, Maria Drummond

**Affiliations:** 1 Edinburgh Medical School, University of Edinburgh, Edinburgh, UK; 2 NHS Grampian, Summerfield House, 2 Eday Road, Aberdeen, AB15 6RE UK; 3 ENRICH Scotland, NHS Research Scotland, Glasgow, UK; 4 Ageing and Health, and Advanced Care Research Centre, Usher Institute, University of Edinburgh, Edinburgh, UK

**Keywords:** Technology, Wearables, Care home, Perceptions, Scoping review

## Abstract

**Purpose:**

This scoping review explores perceptions of care home residents, staff and family members on the use of wearable devices—e.g. smartwatches, fall-detection devices, sensor-enabled clothing—in care homes, on the acceptability, perceived value, and implementation considerations.

**Methods:**

We searched six databases. Two reviewers independently screened studies. Eligible studies reported stakeholders’ perceptions wearable devices used by older adults (65 +)§ in care homes. We followed PRISMA-ScR guidelines. Protocol registered on Open Science Framework (https://doi.org/10.17605/OSF.IO/EJZ3S). Key data were extracted and narratively summarised, then analysed using a deductive and inductive framework based on the research aims.

**Results:**

Of 620 studies, 12 were included. Studies were mostly qualitative and varied in size (*n* = 4–178) using a wide range of wearables: activity/sleep trackers (*N* = 4); wireless monitoring (*N* = 3); incontinence pad sensors (i = 2); posture-improving sensors (*N* = 1); pressure injury prevention (*N* = 1); contact tracing (*N* = 1).Familiarity and aesthetics, including the potential of stigmatisation, influenced acceptability for residents and passive devices were deemed more suitable in cognitive impairment.

Perceived value included reassurance for residents and family members. Devices could improve quality of life. Staff considered the potential impact on workload, and cost as potential implementation considerations.

**Conclusion:**

There is limited literature on wearable device use in care homes, despite the rapid expansion of technology in this setting. Future research should focus on familiar devices that are not stigmatising, and are easy to use, with co-creation approaches and longer evaluation.

## Key summary points

**Aim:**

A scoping review into the perceptions of care home residents, staff and family members on the use of wearable devices.

**Finding:**

Wearable devices are generally acceptable to care home residents, especially when familiar and perceived to bring value. The impact of the wearable device on staff workloads and potential costs is key factors in whether they would support implementation.

**Message:**

Wearable devices could potentially be accepted by care home residents and staff, and future research should co-create ways to implement wearable technologies with residents, families and staff.

## Introduction

People are living longer, and the number of older adults requiring long term care is also rising [1]. Interest has, therefore, grown in how digital technologies might support the health, safety, and well-being of care home residents, in line with global healthcare digitalisation strategies outlined by the WHO [2]. One area of rapid growth is ‘wearable devices’: equipment that can be worn on the body and can monitor multiple parameters and activities [3]. These wearable devices encompass items such as activity trackers and smartwatches, falls-detection technologies, and sensor-embedded garments such as smart vests, belts, or trousers [4].

Wearable devices may offer significant benefits within health and social care as they have the potential to provide real-time physiological and behavioural monitoring [5, 6]. They can also highlight sedentary behaviour, and, with appropriate support, encourage more activity amongst residents and have a potential role in supporting the mental health of this population [7, 8]. Wearable devices may also alleviate workforce pressures by automating routine monitoring tasks and improving care efficiency [9]. Potential barriers to use include lack of perceived value, difficulty implementing into daily life and ease of use [10]. However, existing research has predominantly focussed on technological capabilities and accuracy [11]. Studies which have explored user experiences have largely included community-dwelling older adults who are generally accepting of wearable devices, especially when they feel the device is adding value to their life. Technical support and follow-ups from volunteers may further improve acceptance [10, 12].

The experience of care home residents is likely to differ from people living in their own homes. Typically, care homes vary from private homes in their physical environment and the structured daily routines [13]. This is because they must balance the needs of care home staff and families, as well as residents’ with complex health and care needs, many of whom have cognitive impairment [10, 12]. An estimated 49% of care home residents are living with dementia and it is thought technology could support future care [14]. However, their views are often underrepresented with a reliance on caregivers [14, 15]. Understanding resident perspectives is essential, as acceptability, usability, and perceived value are critical determinants of implementation success [16]. Therefore, this scoping review aims to examine how wearable devices are perceived within care home settings from the perspectives of residents, staff, and family members. Specifically, the review seeks to identify the perceived challenges and opportunities associated with the use of wearable devices and to describe how these perceptions may differ across stakeholder groups. This will highlight considerations to support future implementation.

## Methods

This scoping review was conducted according to a protocol registered on the Open Science Framework (https://doi.org/10.17605/OSF.IO/EJZ3S) and is reported in line with PRISMA-ScR (Preferred Reporting Items for Systematic Reviews and Meta-Analyses extension for Scoping Reviews) guidelines[17] (Appendix 1).

A comprehensive search of PubMed, CINAHL, Scopus, Web of Science, IEEE, and ASSIA was undertaken on 28/07/2025. The PubMed search strategy was developed in collaboration with an academic librarian; we then asked ChatGPT-5 [18] to adapt this search for the six databases. As this is a novel approach, the final strategies were validated by the librarian. Search terms captured concepts relating to wearable technologies, older adults, care homes, and stakeholder perceptions (full strategies are provided in Appendix 2). Due to resource constraints, forward citation searching and author contact were not undertaken.

Titles, abstracts, and full texts were screened in Covidence [19] by two reviewers (ASV and MD), using predefined eligibility criteria.

Studies were included if they:

- Were described as conducted in a care home or nursing home setting
- Involved wearable devices such as smartwatches, fall-detection devices, or sensor-enabled clothing
- Focussed on adults aged 65 years and older who used the devices
- Reported the perceptions of key stakeholders, including residents, staff, or family members.

Studies were excluded if they:

Examined technologies other than wearable devices (e.g. robotics, smart home systems) OR.

Involved only community-dwelling older adults.

No language or date restrictions were applied during screening. Any disagreements were resolved in consultation with a third reviewer (SDS), and consensus was reached for all decisions.

Data extraction was completed independently by ASV and either MD or KH. The Critical Appraisal Skills Programme (CASP) checklist [20] was used to assess the methodological quality of included studies. Consistent with the aims of a scoping review, studies were not excluded on the basis of quality appraisal. Instead, appraisal findings were used to provide context when interpreting the evidence base and to identify methodological strengths and limitations across included studies. A narrative synthesis approach used in a similar review [21] that aimed to understand the what, why and how of using technology with people with dementia was applied to organise and interpret the data. Data relevant to the review aim were coded and combined into overarching themes by ASV and checked by MD to describe stakeholder perceptions of wearable devices in care homes.

## Data analysis

A framework-based inductive and deductive approach [22] was used to synthesise extracted findings. Findings and participant quotations relating to stakeholder perceptions of wearable devices were treated as the unit of analysis. Following familiarisation with the data, an analytic framework was developed deductively from the review aim (acceptability, perceived value, and implementation considerations). It was then iteratively refined through inductive coding of included studies. Data from each study were indexed against the framework and charted into a matrix to enable comparison between stakeholder groups (residents, staff, and relatives). This process supported identification of recurring patterns while maintaining transparency between original study findings and the final synthesis.

## Results

### Overview of included studies

Of 620 papers, 134 were removed as duplicates, leaving 486 which were screened, and of the 46 full-text articles, 12 were included in the final review (Fig. 1). The majority of excluded studies were in the wrong setting, mostly community; studies that were excluded for insufficient data did not include perceptions of stakeholders; or wearable devices were included with other technologies, and it was not possible to isolate evidence on wearable devices. Quality appraisal identified variation in methodological quality across the included studies. Common limitations included small sample sizes, limited reporting of researcher reflexivity, and short evaluation periods. These findings were considered when interpreting the strength and transferability of the review findings.

The included studies (Table 1) used mostly qualitative or mixed methods, and included a wide range of wearable technologies: activity/sleep trackers (*N* = 4); wireless monitoring system (*N* = 3); sensors in incontinence pads (*N* = 2); posture improving sensors (*N* = 1); contact tracing during COVID-19 pandemic (*N* = 1); pressure injury prevention (*N* = 1).

They were mostly from high income countries: UK (N = 3), Europe (*N* = 4) and North and South America (2 USA, 2 Canada, 1 Chile).

Nine papers included the perceptions of residents and staff, and three staff only. The sample sizes (n) were generally small, mostly 10–20 apart from one mixed methods trial with a qualitative component (*n* = 115) [23] and one mixed methods feasibility trial, with only a brief description of perspectives of staff (*n* = 202) [24].

### Synthesis of results

Findings have been clustered around three themes aligned with the review aim: acceptability, perceived value, and implementation considerations and then presented in order of salience (Table 2); a more detailed version can be found in Appendix 3. For residents, acceptability depended on whether devices appeared familiar, dignified, and non-stigmatising. This was of increasing importance for residents with cognitive impairment. For staff, judgments centred on feasibility, including workload impact and cost. Across stakeholder groups, perceived value centred on interpersonal benefits. Family members described reassurance gained through access to information about residents' well-being, residents associated wearable devices with greater safety and quality of life, and staff valued the data provided by wearable devices to support communication and validate care delivery. However, implementation concerns shaped whether these benefits were considered realistic. These included maintenance burden, workflow disruption, and fears of surveillance.

### Acceptability

#### Aesthetics: familiarity and stigma

Several studies mentioned the importance of the aesthetics and familiarity of the wearable device for acceptability [25–27]; for example, watches were widely felt to be acceptable.

Unfamiliar or conspicuous wearable devices were associated with lower acceptance, even when adherence was otherwise high. Wearable devices that did not resemble familiar objects (e.g. watches without a clock face, orthotic-style sensors, access-control bracelets) [26–28] introduced confusion amongst people with advanced dementia and attracted resistance related to visibility and aesthetics. The identification of these wearable devices elicited concerns about stigma, including fears of drawing negative comments or being labelled as a patient [28]. Several residents expressed concerns that visible wearable devices could identify them as patients or attract unwanted attention from others. Participants suggested that smaller devices or those that could be concealed beneath clothing may be more acceptable. Concerns about stigma were most commonly reported in relation to visible bracelets and monitoring devices [26].

#### Cognitive accessibility

Cognitive impairment, including moderate to severe dementia, was common among participants and was not typically reported as a barrier, although this was not always explicitly explored. Higher acceptability was reported of passive wearable devices, such as actigraph bracelets, that required little or no interaction from residents, including those living with cognitive impairment [27]. However, wearable devices that required interaction, interpretation, or self-monitoring were less consistently acceptable in people with cognitive impairment [23].

### Perceived value

#### Remote reassurance

Data collected and interpreted by wearable devices, particularly smartwatches, appeared to provide reassurance to residents regarding their health [26, 29], family members [29], and staff regarding their standards of care [30]. For family members, remote access to wearable-generated health information (such as heart rate and sleep patterns) reassured them that the resident was well. Staff also reported that wearable data offered objective evidence of the care they delivered, which could be useful personally (as validation) and professionally (for families and managers).

#### Safety and quality of life

Wearable devices were found to improve residents’ quality of life by reducing unnecessary wake ups during the night to change pads that were not soiled [31]. They also had the potential to improve safety by allowing residents more access around the home while still being supervised [28]. However, some staff expressed fear of this increasing surveillance of them [30].

#### Information visibility

Wearable devices also had the potential to increase visibility of residents sleep and other health indicators which allowed staff to better understand resident’s behaviour [29]. However, some staff argued that this information was no different to that usually shared across the team in handovers [32]*.*

### Implementation considerations

#### Perceived impact of device on staff workload

Staff perceptions were primarily shaped by the perceived impact of wearable devices on their workload. This impact was mixed and appeared to depend on the degree to which the wearable device aligned with core care tasks. Staff reported reduced workload and improved efficiency when the wearable device directly supported routine care processes such as incontinence monitoring and repositioning prompts [23, 31, 33]. They also reported associated improvements in task coordination and morale. However, two other studies reported negative perceptions [24, 32], particularly increased administrative burden and added responsibility for wearable device management in the absence of clear gains for care delivery. In these cases, wearable devices were primarily resident-centred, such as smartwatches and remote monitoring systems, and did not directly streamline staff tasks. Moreover, staff described the additional handling, charging, and oversight requirements as time-consuming and emotionally burdensome.

#### Financial Implications

Cost was frequently reported as a barrier to implementation [24, 30]. In multi-site care organisations, cost was described as a structural barrier due to the perceived need for uniform deployment across all sites to ensure equity. Upfront costs were cited as limiting adoption and optimal deployment; for example, whole-site monitoring systems intended to support safe wandering were scaled back due to expense, resulting in less targeted alarm systems and more false alerts. Conversely, some studies reported the potential for cost savings, particularly where wearable devices improved resource efficiency [23, 31]. Incontinence sensors that triggered pad changes based on need rather than fixed schedules were noted to reduce unnecessary changes and associated material and labour costs.

#### Exclusion by design

Wearable device acceptability was influenced by alignment between their design and the functional and cognitive characteristics of residents. Inaccurate step counts in participants using mobility aids or with neurological deficits were noted [29]. There were also reported difficulties with self-monitoring due to visual or interface limitations [29]. This indicates that commercially available wearable devices may not be optimised for this population.

## Discussion

### Summary of evidence

In twelve qualitative studies, mostly from high-income Western countries, of the acceptability and perceived value of wearable devices in older adult care homes was not wholly determined by the technology itself but by its alignment with the lived context of residents, families, and staff. Wearable devices were well-received when they were familiar in form, passive in use, and directly supported care tasks and clinical insights; conversely, burdens tied to usability, workload, aesthetics, or financial constraints attenuated uptake even when clinical need or benefit was present. Therefore, findings suggest that implementation success is more likely when wearable devices are designed or deployed when attention is given to the presence of cognitive and functional impairment in the user, local cost structures, and when direct impact on patient and staff workload is clear to allow for appropriate workflow integration.

### Technology and fit within care home life

Technology acceptance frameworks such as the Technology Acceptance Model [34] and Unified Theory of Acceptance and Use of Technology (UTAUT) are commonly used to understand why technologies are adopted, accepted, or rejected [35]. However, existing applications have predominantly focussed on healthcare technologies, including telehealth, electronic health records, remote monitoring, and clinical decision-support systems [36]. Outcomes often prioritise efficiency, clinical effectiveness, and health-related behaviours [36, 37]. While these considerations are important, they may not fully examine the extent to which technology supports quality of life, dignity, autonomy, social relationships, and everyday well-being. Therefore, a broader socio-technical perspective may be required to understand the factors that influence the acceptance and sustained use of wearable devices in care settings.

Residential care settings are defined by routinised time, relational work, and competing pressures such as staffing constraints and regulatory accountability; technologies that work around the rhythms or ease pressure of complex care ecosystems and services are more easily embedded [38]. Those that are perceived to disrupt routines or may introduce additional tasks are resisted [39–41]. Therefore, non-adoption or abandonment of technology by staff and residents may be part of what they consider to be a pragmatic assessment of how well a device “fits” within the care home. The likelihood of acceptance can be maximised by early, in-person training, opportunities for reassurance, and visible support from colleagues or managers to normalise the use of new technologies within existing practice [42, 43]. Without this scaffolding, even promising innovations may fail.

### Supporting sustained use and maximising benefits

Across the included studies, wearable devices supported residents by making health and activity patterns more visible and understandable, which in turn enabled achievable lifestyle changes. Wearable devices can enhance awareness of activity levels, sleep, and physiological indicators in residents and people living with life-limiting conditions, which in turn enables more realistic goal-setting and early recognition of changes in functioning [44–46]. From a behavioural perspective, the mechanisms most consistently associated with benefit include increased self-awareness, reassurance, habit-building, and feelings of control [47–49]. However, the extent to which improved awareness leads to sustained behaviour change remains variable, and motivation may be undermined when residents question the accuracy or personal relevance of the data [50, 51]. Overall, wearable devices may help residents, including people with dementia move toward healthier routines. However, sustained use requires support to use and manage the device and confidence that the feedback they provide is trustworthy and can be integrated into supportive care practices.

This reflects findings in a systematic review of cost-effectiveness of wearable devices in healthcare more generally which found that wearable devices can prove cost-effective in various healthcare scenarios but it depends on the wearable device [52].

### Perspectives of stakeholders

The use of wearable devices in care homes raises important questions about whose values and priorities are centred in decisions about monitoring and safety. This review identified that relatives generally perceived wearable devices as tools that could enhance their sense of reassurance, connection, and oversight regarding a resident’s well-being. Such perceptions align with wider evidence that monitoring technologies are justified through risk reduction and improved quality of care, including where relatives have limited direct visibility of everyday care practices [53]. Indeed, the need for reassurance may lead relatives to favour the use of technology where there is a perceived potential to improve safety, even when this may involve trade-offs related to residents’ privacy, autonomy, or dignity [43], especially when relatives act as proxy decision-makers.

Relatives’ perceptions are further shaped by beliefs about residents technological capability, which can directly influence expectations of adoption and sustained use of wearable devices [54]. Ageing is commonly framed through deficit-based assumptions that associate older age with frailty and technological incompetence [55]. When such assumptions are held by family members, they may inadvertently limit residents’ opportunities to participate in decisions about wearable device use. Therefore, wearable devices can support reassurance and suggest safety for relatives. However, they may risk marginalising residents’ voices if not carefully balanced. Greater co-creation involving residents, relatives, and care staff may help ensure that wearable devices are implemented in ways that respect autonomy and balance the rights and concerns of all stakeholders.

### Considerations for future research

Future research should prioritise studies with larger and more diverse participant groups to better capture the range of experiences and perspectives within care home settings. Future studies should also include longer follow-up and extend beyond the proof-of-concept or pilot studies of developing technologies, including evaluation of both novel and established technologies. It is essential that different organisational, cultural, and care contexts are also considered. In particular, greater effort is needed to directly include care home residents, including those living with cognitive impairment, whose perspectives remain underrepresented, as well as their families or legal representatives. Whilst methodological challenges are acknowledged, innovative approaches such as ethnographic methods, flexible interviewing, and participatory observation may enable more meaningful inclusion. However, it is essential that appropriate ethical and governance structures allow this participation to support improvements in care. Finally, future research on wearable devices’ implementation and evaluation should explore co-creation approaches that actively involve residents, relatives, and care staff from the design stage, and appreciate that implementation of a wearable device in this context is a complex intervention that needs to be done sensitively and with awareness of the context [56].

### Strengths and limitations

A key strength of this scoping review is its focus on the lived context of care homes, bringing together perspectives of residents, relatives, and staff. By synthesising qualitative and mixed-methods evidence, the review captures acceptability as well as social, ethical, and organisational factors shaping implementation. The inclusion of studies involving residents with cognitive impairment strengthens the relevance of the findings to real-world care home populations, where dementia is prevalent. Additionally, the use of a structured and transparent search strategy across multiple databases, with independent review of title, abstract and data extraction from full text supports the rigour and reproducibility of the review.

Several limitations of this scoping review should be acknowledged. First, due to time constraints, the initial stage of thematic analysis was conducted by a single reviewer, which may have resulted in the omission of relevant interpretive insights. However, this was mitigated by checking of the data extraction and themes by an independent reviewer. Second, although multiple major databases from medicine, allied health, engineering and social science were searched, some specialised databases such as PsycInfo and EMBASE were not included. This may have limited the identification of relevant studies. We did not restrict the search by language but did not identify any non-English papers. In addition, we used a Large Language Model [18] to support the translation of search terms. This is a relatively novel approach and may have influenced the final search results, but we mitigated this by checking the final search strategy with a librarian.

The included studies also had limitations, although valuable and rich data is provided in qualitative studies, it is likely that the range of views of residents, families and staff will not have been captured, given the likely diverse physical and cognitive needs and individual preferences. Staff were treated as the major stakeholders in the majority of the studies, which meant our depth of analysis was limited comparatively for the different stakeholder groups. The majority of included studies were conducted in high-income Western countries, and no studies from East Asia were identified despite the substantial development and implementation of digital technologies within countries such as China and Japan. Although no language restrictions were applied, this may reflect differences in publication practices, indexing within the databases searched, or the terminology used to describe wearable technologies and care home settings. Consequently, the findings primarily reflect Western care home contexts and may not fully capture cultural differences in attitudes toward technology, monitoring, privacy, autonomy, and care provision. The transferability of these findings to other geographical and cultural contexts should therefore be considered with caution.

In addition, the results may be biased due to data collection by the researcher, who visited the care homes, provided the wearable devices and, in some cases, also provided technological support. The lack of dedicated support and the need to integrate care of devices into an already-demanding role would likely result in different responses from staff.

## Conclusions

This scoping review found that wearable devices are generally acceptable within care home settings and can provide reassurance by increasing the visibility of health and care-related information. Wearable devices were most positively received when they were familiar in form, passive in use, and clearly aligned with everyday care practices. However, implementation was constrained by concerns about staff workload, device usability and cost. Moreover, there is a risk that technologies prioritising reassurance and monitoring may inadvertently marginalise residents’ rights to privacy and autonomy. These findings suggest that the success of wearable devices in care homes depends less on technical capability and more on their fit within the relational, organisational, and ethical context of care. However, there were only twelve studies identified, with few participants and short duration. Future research should focus on longer-term implementation, greater inclusion of residents’ perspectives and diversity of participants and countries, and co-creative approaches that involve residents, relatives, and care staff. Such approaches may help balance safety and dignity, ensuring that wearable devices support person-centred care rather than inadvertently undermining it.

## Appendix 1

See Table 1.

**Table 1:** Preferred reporting items for systematic reviews and meta-analyses extension for scoping reviews (PRISMA-ScR) Checklist

Section	Item	PRISMA-ScR Checklist item	Reported on page #
Title			
Title	1	Identify the report as a scoping review	Title
Abstract			
Structured summary	2	Provide a structured summary that includes (as applicable): background, objectives, eligibility criteria, sources of evidence, charting methods, results, and conclusions that relate to the review questions and objectives	Abstract
Introduction			
Rationale	3	Describe the rationale for the review in the context of what is already known. Explain why the review questions/objectives lend themselves to a scoping review approach	Introduction
Objectives	4	Provide an explicit statement of the questions and objectives being addressed with reference to their key elements (e.g. population or participants, concepts, and context) or other relevant key elements used to conceptualise the review questions and/or objectives	Introduction
Methods			
Protocol and registration	5	Indicate whether a review protocol exists; state if and where it can be accessed (e.g., a Web address); and if available, provide registration information, including the registration number	Methods
Eligibility criteria	6	Specify characteristics of the sources of evidence used as eligibility criteria (e.g. years considered, language, and publication status), and provide a rationale	Methods
Information sources*	7	Describe all information sources in the search (e.g. databases with dates of coverage and contact with authors to identify additional sources), as well as the date the most recent search was executed	Methods
Search	8	Present the full electronic search strategy for at least 1 database, including any limits used, such that it could be repeated	Methods
Selection of sources of evidence†	9	State the process for selecting sources of evidence (i.e. screening and eligibility) included in the scoping review	Methods
Data charting process‡	10	Describe the methods of charting data from the included sources of evidence (e.g. calibrated forms or forms that have been tested by the team before their use, and whether data charting was done independently or in duplicate) and any processes for obtaining and confirming data from investigators	Methods
Data items	11	List and define all variables for which data were sought and any assumptions and simplifications made	Methods
Critical appraisal of individual sources of evidence§	12	If done, provide a rationale for conducting a critical appraisal of included sources of evidence; describe the methods used and how this information was used in any data synthesis (if appropriate)	Methods
Synthesis of results	13	Describe the methods of handling and summarising the data that were charted	Methods
Results			
Selection of sources of evidence	14	Give numbers of sources of evidence screened, assessed for eligibility, and included in the review, with reasons for exclusions at each stage, ideally using a flow diagram	Results
Characteristics of sources of evidence	15	For each source of evidence, present characteristics for which data were charted and provide the citations	​​Results​
Critical appraisal within sources of evidence	16	If done, present data on critical appraisal of included sources of evidence (see item 12)	Results
Results of individual sources of evidence	17	For each included source of evidence, present the relevant data that were charted that relate to the review questions and objectives	Results
Synthesis of results	18	Summarise and/or present the charting results as they relate to the review questions and objectives	Results
Discussion			
Summary of evidence	19	Summarise the main results (including an overview of concepts, themes, and types of evidence available),link to the review questions and objectives, and consider the relevance to key groups	Discussion
Limitations	20	Discuss the limitations of the scoping review process	Discussion
Conclusions	21	Provide a general interpretation of the results with respect to the review questions and objectives, as well as potential implications and/or next steps	Discussion
Funding			
Funding	22	Describe sources of funding for the included sources of evidence, as well as sources of funding for the scoping review. Describe the role of the funders of the scoping review	Abstract

*JBI* Joanna Briggs Institute; *PRISMA-ScR* Preferred Reporting Items for Systematic reviews and Meta-Analyses extension for Scoping Reviews.

* Where *sources of evidence* (see second footnote) are compiled from, such as bibliographic databases, social media platforms, and Web sites.

† A more inclusive/heterogeneous term used to account for the different types of evidence or data sources (e.g. quantitative and/or qualitative research, expert opinion, and policy documents) that may be eligible in a scoping review as opposed to only studies. This is not to be confused with *information sources* (see first footnote).

‡ The frameworks by Arksey and O’Malley (6) and Levac and colleagues (7) and the JBI guidance (4, 5) refer to the process of data extraction in a scoping review as data charting.

§ The process of systematically examining research evidence to assess its validity, results, and relevance before using it to inform a decision. This term is used for items 12 and 19 instead of "risk of bias" (which is more applicable to systematic reviews of interventions) to include and acknowledge the various sources of evidence that may be used in a scoping review (e.g. quantitative and/or qualitative research, expert opinion, and policy document).

## Appendix 2: Search strategies

### PubMed

(((wearable* [tiab] OR wearable device* [tiab] OR fall detector [tiab] OR smartwatch* [tiab] OR accelerometer [tiab] OR pedometer [tiab] OR wearable technology [tiab] OR wearable sensor [tiab] OR wireless sensor [tiab] OR movement sensor [tiab] OR activity tracker [tiab] OR wearable health devices [tiab] OR activity tracker* [tiab] OR biometric sensor [tiab] OR Digital health [tiab] OR wearable electrical device* [mesh] OR fitness tracker* [mesh]) AND (care home* [tiab] OR residential home* [tiab] OR nursing home [tiab] OR assisted living facility [tiab] OR long term care facility [tiab])) AND (elderly [tiab] OR aged [mesh] OR over 80 [tiab] OR aging [tiab] OR older adults [tiab])) AND (perspective* [tiab] OR perception* [tiab] OR acceptability [tiab] OR usability [tiab] OR feasibility [tiab] OR focus group [tiab] OR semi-structured interviews [tiab] OR qualitative [tiab] OR qualitative review [tiab]).

### CINAHL

((wearable* OR "wearable device*" OR "fall detector" OR smartwatch* OR accelerometer OR pedometer OR "wearable technology" OR "wearable sensor" OR "wireless sensor" OR "movement sensor" OR "activity tracker" OR "wearable health devices" OR "activity tracker*" OR "biometric sensor" OR "digital health" OR "wearable electrical device*" OR "fitness tracker*").

AND

("care home*" OR "residential home*" OR "nursing home" OR "assisted living facility" OR "long term care facility").

AND

(elderly OR aged OR "over 80" OR aging OR "older adults").

AND

(perspective* OR perception* OR acceptability OR usability OR feasibility OR "focus group" OR "semi-structured interviews" OR qualitative OR "qualitative review")).

### Scopus

(TITLE-ABS-KEY(wearable* OR "wearable device*" OR "fall detector" OR smartwatch* OR accelerometer OR pedometer OR "wearable technology" OR "wearable sensor" OR "wireless sensor" OR "movement sensor" OR "activity tracker" OR "wearable health devices" OR "activity tracker*" OR "biometric sensor" OR "digital health" OR "wearable electrical device*" OR "fitness tracker*")).

AND

(TITLE-ABS-KEY("care home*" OR "residential home*" OR "nursing home" OR "assisted living facility" OR "long term care facility")).

AND

(TITLE-ABS-KEY(elderly OR aged OR "over 80" OR aging OR "older adults")).

AND

(TITLE-ABS-KEY(perspective* OR perception* OR acceptability OR usability OR feasibility OR "focus group" OR "semi-structured interviews" OR qualitative OR "qualitative review")).

### Web of Science

(TS = (wearable* OR "wearable device*" OR "fall detector" OR smartwatch* OR accelerometer OR pedometer OR "wearable technology" OR "wearable sensor" OR "wireless sensor" OR "movement sensor" OR "activity tracker" OR "wearable health devices" OR "activity tracker*" OR "biometric sensor" OR "digital health" OR "wearable electrical device*" OR "fitness tracker*")).

AND

(TS = ("care home*" OR "residential home*" OR "nursing home" OR "assisted living facility" OR "long term care facility")).

AND

(TS = (elderly OR aged OR "over 80" OR aging OR "older adults")).

AND

(TS = (perspective* OR perception* OR acceptability OR usability OR feasibility OR "focus group" OR "semi-structured interviews" OR qualitative OR "qualitative review")).

### IEE

(("wearable" OR "wearable device" OR "fall detector" OR "smartwatch" OR "accelerometer" OR "pedometer" OR "wearable technology" OR "wearable sensor" OR "wireless sensor" OR "movement sensor" OR "activity tracker" OR "wearable health devices" OR "biometric sensor" OR "digital health" OR "wearable electrical device" OR "fitness tracker").

AND

("care home" OR "residential home" OR "nursing home" OR "assisted living facility" OR "long term care facility").

AND

(elderly OR aged OR "older adults" OR aging OR "over 80").

AND

(perspective OR perception OR acceptability OR usability OR feasibility OR "focus group" OR "semi-structured interviews" OR qualitative OR "qualitative review")).

### ASSIA

(wearable* OR "wearable device*" OR "fall detector" OR smartwatch* OR accelerometer OR pedometer OR "wearable technology" OR "wearable sensor" OR "wireless sensor" OR "movement sensor" OR "activity tracker" OR "wearable health devices" OR "activity tracker*" OR "biometric sensor" OR "digital health" OR "wearable electrical device*" OR "fitness tracker*").

AND

("care home*" OR "residential home*" OR "nursing home" OR "assisted living facility" OR "long term care facility").

AND

(elderly OR aged OR "over 80" OR aging OR "older adults").

AND

(perspective* OR perception* OR acceptability OR usability OR feasibility OR "focus group" OR "semi-structured interviews" OR qualitative OR "qualitative review").

## Appendix 3: Overview of thematic framework, themes and illustrative data

See Table 2.

**Table 2:** Summary of key themes with sources and illustrative quotes

Theme	Analytic Code	Primary stakeholder(s)	Example of evidence
Acceptability	Familiarity and normalisation [25, 27, 29]	Residents	“a watch is such a familiar item that even in late-stage dementia the lack of a watch face is recognised as an anomaly and contributes to reduced compliance” Observation from researcher[27] “*I used to wear a watch all the time*.” Resident [29] “No resistance [to wearing a sensor around the waist]. The patient was already using a pouch around the waist.” Observation from researcher [25]
	Stigma and identity [26, 28, 31]	Residents	*“Well, it’s complicated because the jokes would start, the negative comments and all that, and here it’s difficult…”* Resident [26] And wearing a bracelet yourself? *“I just do not like it all … They’d better not do that with me—then everybody will know you belong to something … like a patient ….”* Resident [28] When asked about the appearance of the [continence] sensor *“Oval and not too big … even if you wear it on your belly* … [expression of shame].”Resident [31]
	Cognitive accessibility [23, 28, 33]	Residents; Staff	*“…some of them are not really favorable for like people that are very cognitively impaired … … some of them really play with it or some of them are not really conscious about it … sometimes, they remove their clothes so the sensors might get lost ….*” Staff [23] [Bracelet allowed for more freedom in the nursing home which could cause distress] One resident was found shouting “I’m lost, I’m lost” for 15 min Observation from researcher [28] *Sensors became detached due to self-soothing behaviours amongst residents with dementia* Staff [33]
Perceived value	Reassurance [23, 26, 29]	Families; Residents; staff	*“When I’m walking, it (the device) helps me to stay on my feet and I feel like I’m walking okay. I go out a lot, I’m 84 years old, I take the bus, I take the subway, and I would like to be sure that I’m not going to fall over or that I won’t be knocked down”* Resident [26] *good if I could help watch things like her heart rate and sleep at home*.” Family member [29] *“… that report showed us that her outputs showed she didn’t always need to be changed. It was good for us because [the unit manager] got to see it so it makes it like I am doing my job. So I guess it just verifies that she is being looked after and she has behavior issues.”* Staff [23]
	Safety and quality of life [23, 28, 31]	Residents; Staff	*It’s a shame to wake up residents at night and to be confronted with a diaper that is not saturated. The residents are awake and their sleep interrupted … if we can prevent this then we should consider this technology yes*.” Staff [31] “*There was one lady who was very agitated with wearing the night brief and we could never figure out why she was so agitated and then we started talking to her about wearing the brief and she said, ‘I’m so hot down there.’ And then we came together as a team and figured out that yeah if you look at her voiding report, she doesn’t warrant it and we could just use a little pad so she won’t be as hot … she was a lot more comfortable afterwards.” Staff* [23] [Bracelet which allows access to more parts of the nursing home allowed the resident to have more freedom around the nursing home] *Night nurse said resident “got out of bed less since she was able to wander during the day”* Staff [28]
	Information visibility [29, 32]	Staff	[smartwatch helps therapists understand if] “*residents were not cooperating with therapy because they didn't sleep well*.” Staff [29] *“Generally, our communication systems around our hand over are that…those type of things are our main highlighters. In terms of a hand over, [it] would be, “Did they sleep well?” “Did they have any falls today?” “Did they eat?” “Did they eat well?” “Are they drinking well?” All that type of thing is basic. [So we are] doing that anyway.* Staff [32]
Implementation considerations	Workload and workflow fit [23, 24, 31–33]	Staff	Decreased workload *“Yeah I see some benefits … You could know better when to change the diaper… At the moment we work with scheduled diaper checks, but sometimes … the diaper is not saturated. However, we know we cannot wait until the next scheduled diaper check, we cannot force them [residents] to pee on command, and we don’t have time to go more than planned. So we change the diaper regarding the saturation.”* Staff [31] *“To know when the residents are going to the bathroom and when they need to be changed and just giving that output of timing. So I think that’s a really big advantage. When our time is so restricted to everything, having something like that to give us that output that we can use throughout our shift really makes a difference.”* Staff [23] Increased workload *“…It is time-consuming. By the time you go to the resident, get the watch off them, go up, plug it in…you get called on the way to do other things. And you have to put the residents’ needs before charging a watch, you know?”* Staff[32]
	Resource and surveillance concerns [23, 30]	Staff; Organisations	*“… that report showed us that her outputs showed she didn’t always need to be changed. It was good for us because* [the unit manager] *got to see it so it makes it like I am doing my job. So I guess it just verifies that she is being looked after and she has behavior issues*.” Staff [23] Staff felt they were being monitored *“big brother tool for management*” – not substantiated Staff (Hall A., 2019 #11
	Excluded by design [7, 24, 26, 29]	Resident	*“Resident #4 was frustrated and expressed disappointment as she could only read the time due to the small font, which prevented self-monitoring”* Resident [29] “adaptations such as putting devices in handbags or on walking frames and wheelchairs potentially compromise BLE (bluetooth low energy) signals, performance and reliability.” Observation from researcher [24]

Italics = direct quotes from participants, non-italic = observation of researcher

## Declarations

### Data availability

The data underlying this study are available in the findings section and online supplementary material.
